# Supplementary material for: Myofascial release versus Mulligan sustained natural apophyseal glides’ immediate and short-term effects on pain, function, and mobility in non-specific low back pain
Source: PeerJ. 2021 Mar 15;9:e10706. doi: 10.7717/peerj.10706 (PMC7971081; doi:10.7717/peerj.10706)
Supplement: Supplemental Information 2 [file peerj-09-10706-s002.docx]

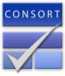


CONSORT CHECKLIST 2010

| **Item** | **Description** | **Reported on line number** |
| --- | --- | --- |
| Title | Identification of study as randomized pilot or feasibility trial | 1 |
| Authors ^[[1]](#footnote-1)^ | Contact details for the corresponding author | 14-19 |
| Trial design | Description of pilot trial design (eg, parallel, cluster) | 30 |
| Methods |  |  |
| Participants | Eligibility criteria for participants and the settings where the pilot trial was conducted | 136-144 |
| Interventions | Interventions intended for each group | 171-197 (Figure 2&3) |
| Objective | Specific objectives of the pilot trial | 117-121 |
| Outcome | Prespecified assessment or measurement to address the pilot trial objectives^[[2]](#footnote-2)^ | 147-170 |
| Randomization | How participants were allocated to interventions | 130-132 |
| Blinding (masking) | Whether or not participants, care givers, and those assessing the outcomes were blinded to group assignment | 132-134, 148, 205 |
| Results |  |  |
| Numbers randomized | Number of participants screened and randomised to each group for the pilot trial objectives** | 219-222 / Figure 1 |
| Recruitment | Trial statust | Completed |
| Numbers analysed | Number of participants analysed in each group for the pilot objectives** | 221-224/ Figure1 |
| Outcome | Results for the pilot objectives, including any expressions of uncertainty** | 226-238 |
| Harms | Important adverse events or side effects | No adverse event observed |
| Conclusions | General interpretation of the results of pilot trial and their implications for the future definitive trial | 310-314 |
| Trial registration | Registration number for pilot trial and name of trial register | 126-128 |
| Funding | Source of funding for pilot trial | PeerJ section |

1. [↑](#footnote-ref-1)
2. [↑](#footnote-ref-2)
