# Supplementary material for: Myofascial release versus Mulligan sustained natural apophyseal glides’ immediate and short-term effects on pain, function, and mobility in non-specific low back pain
Source: PeerJ. 2021 Mar 15;9:e10706. doi: 10.7717/peerj.10706 (PMC7971081; doi:10.7717/peerj.10706)
Supplement: Supplemental Information 3 [file peerj-09-10706-s003.pdf]

Introduction

Low back pain (LBP) is the leading cause of years lived with disability in both developed and developing countries.[1] Low back pain is considered as a debilitating musculoskeletal health condition, ranked first in terms of disease burden worldwide.[2] The global age-standardised point prevalence of LBP (from 0 to 100 years of age) in 2010 was estimated to be 9.4%, the mean point prevalence of LBP was estimated to be 18.3%, and one month prevalence was 30.8%.[3]

Low back pain is defined as pain in the area on the posterior aspect of the body from the lower margin of the twelfth ribs to the lower gluteal folds with or without pain referred in to one or both lower limb.[3,4] Low back pain is broadly classified as specific LBP and nonspecific LBP (NSLBP).[1,3,4] A known identifiable pathology is called as specific LBP and NSLBP is back pain of unknown underlying pathology which is characterised by pain, muscle tension and stiffness.[1,4] Nonspecific LBP severely limits activities of daily living, induce substantial medical consumption resulting in prolonged disability.[1,2] Low back pain can be categorized in to, acute LBP which lasts for less than 6 weeks, sub-acute LBP lasts for between 6 weeks and 3 months and chronic LBP has a duration of more than 3 months.[4]

The proposed pathomechanics of NSLBP attributed to changes in lumbosacral proprioception, core muscle recruitment patterns, macroscopic degeneration of lumbar stabilisers, abnormal tissue loading and weakness of both gluteal and paraspinal muscles. The evident depletion of lumbar motor control in NSLBP leads to uncontrolled movements, this altered abnormal movement pattern increases tissue stiffness which stresses lumbar spine, causing pain exacerbation.[5]

The primary management being pain reduction, NSLBP also focuses on patient education, analgesic medication, non-medical therapy and timely review.[6] Non-medical

physical and rehabilitation interventions include Manual therapy,[7] back schools, exercises, electrical modalities and multidisciplinary rehabilitation.[6]

Manual therapy is forms of hands-on soft tissue or joint mobilization techniques to modulate pain and also improve extensibility of contractile tissues and improve restricted movement of joints.[7] Manual therapies such as Mulligan mobilization, Maitland mobilization, myofascial release therapy are used routinely in clinical practice and have shown high evidence of its effectiveness of the treatment in NSLBP.[8]

The Mulligan concept is based on the minor position faults of articulating surfaces of joint following injury or strain resulting in painful and restricted range of motion (ROM).[7,8] Mulligan concept includes natural apophyseal glides (NAGs), sustained natural apophyseal glides (SNAGs) and mobilizations with movement (MWMs).[7,9] Sustained natural apophyseal glides is a technique that involves application of passive accessory glide parallel to the joint plane using the spinous process or transverse process of the vertebra while the subject simultaneously executes an active movement which was previously painful or restricted.[7–10]

Two studies were found and replicated in a systematic review among NSLBP, one of the study results showed added effects of SNAGs to conventional therapy improved pain perception and function.[7,8,10] The study also showed the treatment had an immediate and short term improvement in lumbar flexion ROM among NSLBP[7,10] and healthy individuals.[11] In another study, SNAGs found to improve lumbar flexion ROM when SNAGs and McKenzie extension exercises were compared among chronic mechanical LBP populations.[12]

Myofascial release (MFR) is a manual therapy treatment that involves guided low load, long duration mechanical forces to manipulate the myofascial complex, aiming to restore optimal muscle length, decrease pain, and improve function as it is effective to provide immediate relief of pain and tissue tenderness.[13,14]

MFR as an adjunct to specific back exercises (SBE) showed superior results than exercise alone in LBP.[13,14] MFR also found to be effective in improving pain perception, releasing impaired sliding fascial mobility[13,15] and functional abilities[16] among nonspecific neck pain (NSNP) and NSLBP.

In 2018 Rezkallah et.al found significant improvement in ROM, pain reduction and neck disability on comparing the effects of MFR and SNAGs in NSNP.[17] Hence there is a need to study same effects of MFR and SNAGs among NSLBP populations.

Review of literature

Studies on Sustained Natural Apophyseal Glides (SNAGs)

Hisham Mohamed Hussien et.al conducted a study to determine the added effects of lumbar SNAGs to a conventional physical therapy on chronic NSLBP. 42 participants were randomly divided into the study group (n=23) who received a conventional physical therapy (stretching and strengthening exercises) and SNAGs and the control group (n=19) received only conventional physical therapy 3 times per week for 1 month. The study showed that added SNAGs to conventional physical therapy in the treatment of chronic NSLBP result in greater improvement of repositioning error, pain relief, and improved function.[7,8]

Benjamin Hidalgo et.al conducted a randomized placebo trial on NSLBP subjects to compare the immediate and short-term effects of lumbar SNAGs and to 2 new kinematic algorithms for ROM (KA-R) and speed. 32 participants were randomized into a real-SNAG group and a sham-SNAG group. All patients were treated for a single session with flexion SNAGs for 3 sets of 6 repetitions each to the lumbar spine in sitting position. This study showed evidence that lumbar spine SNAGs had a short-term favourable effect on KA-R, pain, and function in patients with NSLBP.[10]

Maria Moutzouri et.al conducted a study to determine the effects of the SNAGs in the lumbar flexion range on asymptomatic subjects. 49 asymptomatic subjects were randomly assigned to receive either SNAG mobilisation (n = 25), or a sham mobilisation (n = 24). Three sets of 10 repetition were given in both the groups. The authors concluded that SNAG mobilisation and sham mobilisation showed to have equal and effective lumbar flexion ROM when measured using three dimensional electronic goniometer (Zebris CMS20).[11]

Waqqar S et.al conducted a study to compare McKenzie Extension Exercises (MEE) and SNAGS for chronic mechanical LBP. 37 subjects delivered intervention for 4 weeks (2

session per week and 1 session per day) of intervention. The authors concluded that MEE are clinically slightly more effective in the management of pain and disability, while SNAGs are more effective in the improvement of lumbar ROM for individuals with chronic Mechanical LBP.[12]

Studies on Myofascial Release (MFR)

M.S. Ajimsha et.al conducted a study on effectiveness of Myofascial release in the management of chronic LBP in nursing professionals. The intervention for MFR group and sham MFR group consisted of 24 sessions over 8 weeks. The patients in the MFR group reported a 53.3% and 29.7% decrease in pain on McGill Pain Questionnaire (MPQ) and disability score on Quebec Back Pain Disability Scale (QBPDS) respectively compared to patients in the control group, who reported 26.1% and 9.8% of improvement. Hence this study showed an evidence that MFR when used as an adjunct to SBE is more effective than a control group with SBE for chronic LBP.[13,14]

Tozzi .P et.al studied pain perception and the mobility of fascial layers by using a dynamic ultrasound (US) in patients with NSNP and NSLBP. 60 patients each with NSNP and NSLBP were divided into experimental and control groups. The results found that MFR can improve impaired fascial mobility when analysed using US, and also pain perception over a short term duration in NSNP and NSLBP compared sham control group.[13,15]

Sweta V. Gauns et.al done a study to compare gross MFR of upper limb and neck against routine conventional therapy in mechanical NP. 40 patients were allocated to experimental and control group. Conventional therapy included with moist pack, TENS and stretching and strengthening exercise. The study results showed gross MFR is an effective technique for subjects with mechanical NP and has a faster rate of improvement than the control group.[16]

Studies on MFR and Mulligan SNAGs

Sohier S. Rezkallah et.al conducted a study to compare SNAG's and MFR combined with exercises in NSNP. 70 individuals were allocated randomly into 3 groups, SNAG's group, MFR group and the control group. The selected common exercises to all 3 groups included stretching and strengthening of the posterior neck muscles, and neck straightening exercises for 12 sessions, 3 sessions per week for 4 consecutive weeks. SNAG's and MFR group yielded significant reduction in pain and neck disability, and increase in neck ROM compared to control group.[17]

Studies on outcome measures

Rahim Sadeghi et.al conducted a study to assess the Reliability of Bubble Inclinometer and Tape measure in determining lumbar spine ROM in healthy individuals and chronic NSLBP patients and the results found that intraclass correlation coefficients and standard error of measurement was 0.770–0.982 and 0.38–1.20 respectively.[18]

Cheryl Hefford et.al conducted a study to determine Validity and Reliability of the Patient-Specific Functional Scale results suggested of moderate to good reliability with an Intraclass coefficient of 0.713 and minimal important difference (MID) was 1.2.[19]

Fritz J et.al conducted a comparative study on Modified Oswestry Low Back Pain Disability Questionnaire and the Quebec Back Pain Disability Scale and results showed intraclass correlation coefficient (ICC) of 0.90, minimum clinically important difference (MCID) ranging between 0.82 to 0.99.[20]

Gillian A. Hawker et.al conducted study on reliability and validity of VAS on pain and the results showed good test–retest reliability higher among literate ($r= 0.94$, $p< 0.001$) than illiterate ($r= 0.71$, $p<0.001$). The correlation between vertical and horizontal orientations of the

VAS is 0.99. The construct validity showed to be highly correlated with 5-point verbal descriptive scale with correlations ranging from 0.71–0.78.[21]

Knowledge gap identified

There was only one study which had short term effects on pain, ROM and functional abilities on comparing SNAGs and MFR in NSNP. Therefore the study intends to find similar effects on NSLBP population on comparing SNAGs and MFR.

Aim of the study

To compare the effects of myofascial release and sustained natural apophyseal glides among nonspecific low back pain

Objectives of the study

- To compare the immediate and short term effects of MFR and Mulligan SNAGS on pain using Visual Analog Scale (VAS)
- To compare the immediate and short term effects of MFR and Mulligan SNAGS using Patient Specific Function Scale (PSFS)
- To determine the immediate and short term improvement in lumbar range of motion using bubble inclinometer
- To find the effects of MFR and Mulligan SNAGS on disability using Oswestry Disability Index(ODI)

Implications

If either MFR or SNAGs is proven to be superior over the other, it can be used independently or as an adjunct with the conventional therapy to improve pain perception, increase lumbar ROM and decrease disability level among NSLBP patients for long lasting effects of treatment.

Methodology

Study setting: - KMC Hospital (B.R Ambedkar circle and Attavara) Mangaluru

Study design: - Randomized clinical trial

Study participants: - subjects with nonspecific low back pain

Sampling method: Block randomisation- Subjects will be divided into subgroup called blocks in which a set of 6 blocks will have 4 combination each. The subjects in each block will be randomly allocated into either SNAGs or MFR group will be enclosed in a concealed opaque envelope.

Tester

A qualified physiotherapist pursuing his master's degree in Physiotherapy at KMC Mangaluru will conduct the study under the guidance of Assistant professors, Department of Physiotherapy Mangaluru

Assessor

An independent assessor who is a qualified physiotherapist at KMC, Mangaluru, blinded from allocated treatment groups will measure the pre and post intervention outcomes of the study participants.

Inclusion criteria

- Subjects with nonspecific low back pain
- Sub-acute, chronic
- Age group between 18 to 60 years
- VAS ≥ 4

Exclusion criteria

- Disc hernia, radiculopathy
- Spinal pathology (fracture or tumors) or history of any spinal surgery
- Lumbar canal stenosis
- Scoliosis, kyphosis, osteoporosis

Sample size

$$n = 2(Z_{1-\alpha} + Z_{1-\beta})^2 \sigma^2 / d^2$$

$Z_{1-\alpha}$= 1.96 at 95% confidence interval

$Z_{1-\beta}$=0.84 at 80% power

Combined standard deviation, σ=0.8

d=0.54

Sample size, n=34 in each group[17]

Study duration December 2018 to March 2020

Materials

- Mulligan Belt
- Bubble inclinometer
- Skin marking pen

Procedure

The study protocol will be submitted to the Scientific Committee and Institutional Ethics Committee of KMC Mangaluru, Manipal Academy of Higher Education (MAHE) for approval. Upon approval, the subjects referred or non-referred by Orthopedician to the Department of Physiotherapy will be approached. The informed consent will be taken from the willing subjects and the purpose of the study will be explained. Post screening for inclusion criteria, eligible subjects will be allocated into two either SNAGs or MFR group by block randomisation. Demographic data and baseline data for VAS, PSFS, ODI and ROM will be collected.

Procedure for SNAGs

SNAGs will be administered based on the clinical presentation of the patient. ASIS of patient will be stabilized by the therapist using a belt around his hip joint. The ulnar border of the hand will be placed according to the pain presentation i.e. for Flexion range restriction the hand will be placed inferior to spinous process of vertebrae to involved segment where for extension hand will be placed superior to the spinous process of vertebrae. Thumb is placed over transverse process for unilateral presentation of pain. The patient then asked to perform the movement which elicits pain and backs off, then the therapist applies a parallel passive accessory glide to facet joint plane over the segment and patient will be asked to perform the movement which was painful before and then return to the starting position while the therapist maintain his mobilizing force until the end. If the movement is pain free throughout the range then therapist is on correct level if not then change the level. The glides will be applied 6 repetitions for 3 sets to improve the ROM and reduce pain.

Procedure for MFR

A gross stretch (MFR) will applied over the poster aspect of the body and the traction will be maintained to hold the tissue at its end range at least 90 to 120 seconds before the tissue will begin to soften and lengthen, The stretch will be held until the therapist feels giving way of taut tissue.

Procedure to measure outcomes

To measure pain, the patient will be administered 100mm Visual Analog Scale (VAS) and asked to mark a point on the scale based on his/her pain intensity, The VAS score for pain will be measured from left hand end of line to point patient marks.

To measure functional ability, the patient will be asked to write down the 3 activities which restrict or unable to do routine activity and will be asked to rate score between "0 to 10" for each activity where 0 is unable to perform/difficult to do and 10 is able to do as before.

To measure disability level, the patient will be given with the questionnaire and asked to tick appropriate option in all the 10 sections. This questionnaire has been designed to give information how low back or leg pain is affecting ability to manage in everyday life.

To check ROM, mark the spinous processes of the T12 and S2 vertebrae using a skin marking pencil, with the patient in the standing position. Place one inclinometer over the spinous process of T12 and the second inclinometer over the midline of the sacrum at S2. Then zero both inclinometers. For flexion ask the subject to bend backward as far as possible and for extension ROM ask the subject to bend backward as far as possible. To measure lateral flexion range of motion, ask the subject to bend the trunk laterally while keeping both feet flat on the ground and the knees straight. Maintain the inclinometers firmly against the spine during the motion. Read and record the degrees from both inclinometers at the end of the motion. Subtract

the degrees on the sacral inclinometer from the degrees on the T12 inclinometer to obtain the lumbar flexion and extension and lateral flexion ROM respectively.

Core strengthening exercises (lumbar multifidus and transverse abdominis) and gluteal muscle strength training and ergonomic advices about posture and lifting techniques will be given to incorporate regularly at home.

The outcome measures will be taken from patients immediately after the treatment and final readings will be taken on the last day of the follow-up.

Outcome variables: - Pain, Range of motion, Disability, Functional status

Data analysis:-

- Data will be analyzed using SPSS version 17.0
- Student's unpaired t-test will be used between the groups
- ANOVA with Post-Hoc test and Bonferroni's t-test will be applied across the groups
- p value <0.05 will be considered as statistically significant

Data collection tools: -

- Visual Analog Scale (VAS)
- Patient Specific Function Scale (PSFS)
- Bubble Inclinometer
- Modified Oswestry Disability Index (modified ODI)

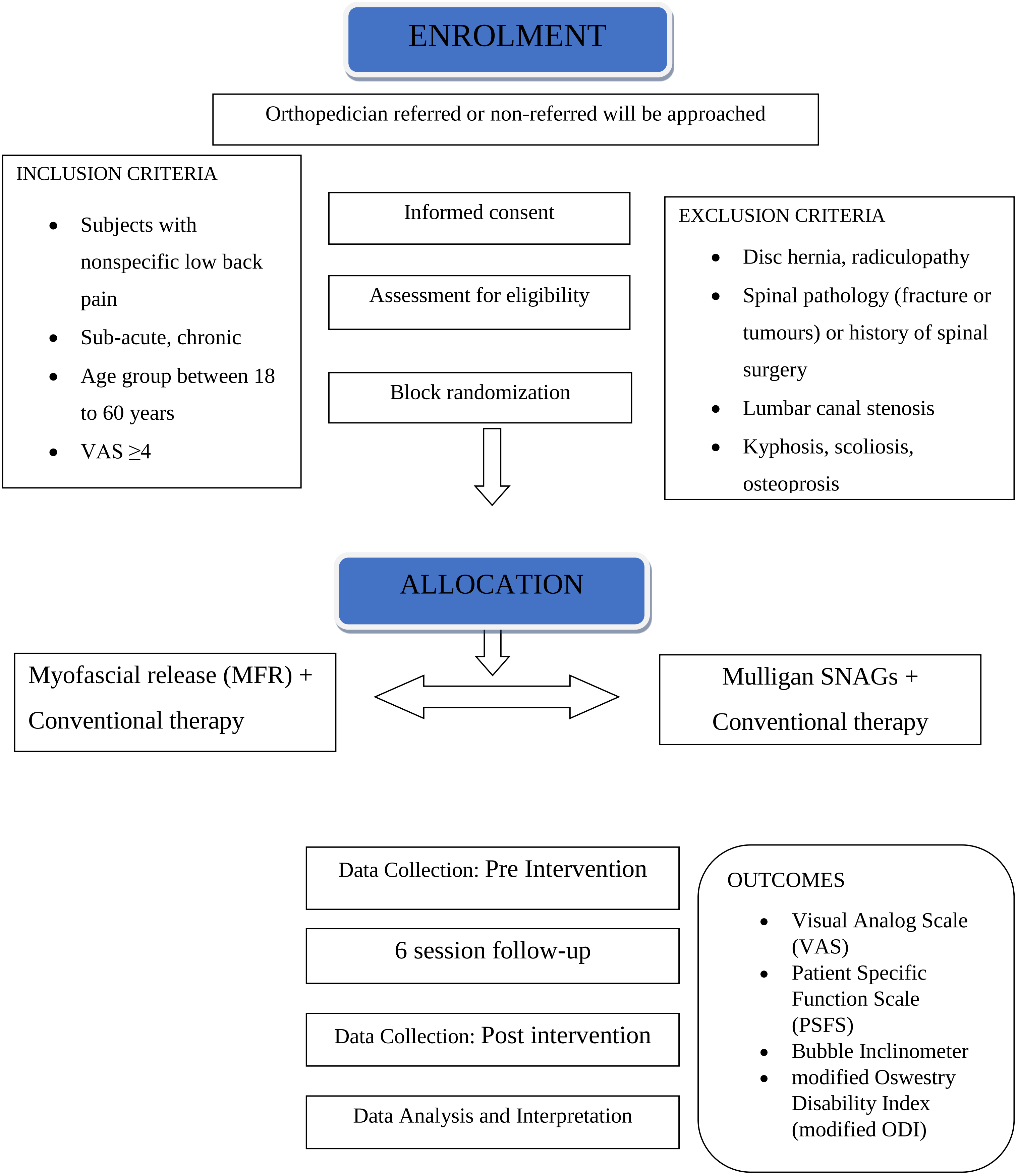
ENROLMENT
Orthopedician referred or non-referred will be approached
INCLUSION CRITERIA
Subjects with nonspecific low back pain
Sub-acute, chronic
Age group between 18 to 60 years
VAS ≥4
Informed consent
Assessment for eligibility
Block randomization
EXCLUSION CRITERIA
Disc hernia, radiculopathy
Spinal pathology (fracture or tumours) or history of spinal surgery
Lumbar canal stenosis
Kyphosis, scoliosis, osteoprosis
ALLOCATION
Myofascial release (MFR) + Conventional therapy
Mulligan SNAGs + Conventional therapy
Data Collection: Pre Intervention
6 session follow-up
Data Collection: Post intervention
Data Analysis and Interpretation
OUTCOMES
Visual Analog Scale (VAS)
Patient Specific Function Scale (PSFS)
Bubble Inclinometer
modified Oswestry Disability Index (modified ODI)

References

1. Maher C, Underwood M, Buchbinder R. Non-specific low back pain. *Lancet*. 2017;389(10070):736–47.

2. Vos T, Abajobir AA, Abbafati C, Abbas KM, Abate KH, Abd-Allah F, et al. Global, regional, and national incidence, prevalence, and years lived with disability for 328 diseases and injuries for 195 countries, 1990-2016: A systematic analysis for the Global Burden of Disease Study 2016. *Lancet*. 2017;390(10100):1211–59.

3. Hoy D, March L, Brooks P, Blyth F, Woolf A, Bain C, et al. The global burden of low back pain: Estimates from the Global Burden of Disease 2010 study. *Ann Rheum Dis*. 2014;73(6):968–74.

4. Krismer M, van Tulder M. Low back pain (non-specific). *Best Pract Res Clin Rheumatol*. 2007;21(1):77–91.

5. Goubert D, Van Oosterwijck J, Meeus M, Danneels L. Structural changes of lumbar muscles in non-specific low back pain. *Pain physician.* 2016;19(7):985-99.

6. Van Middelkoop M, Rubinstein SM, Kuijpers T, Verhagen AP, Ostelo R, Koes BW, et al. A systematic review on the effectiveness of physical and rehabilitation interventions for chronic non-specific low back pain. *Eur Spine J*. 2011;20(1):19–39.

7. Pourahmadi MR, Mohsenifar H, Dariush M, Aftabi A, Amiri A. Effectiveness of mobilization with movement (Mulligan concept techniques) on low back pain: a systematic review. *Clin Rehabil*. 2018;

8. Hussien HM, Abdel-Raoof NA, Kattabei OM, Ahmed HH. Effect of Mulligan Concept Lumbar SNAG on Chronic Nonspecific Low Back Pain. *J Chiropr Med*. 2017;16(2):94–102.

9. Wilson E. The Mulligan concept: NAGS, SNAGS and mobilizations with movement. *J Bodyw Mov Ther*. 2001;5(2):81–9.

10. Hidalgo B, Pitance L, Hall T, Detrembleur C, Nielens H. Short-Term Effects of Mulligan Mobilization With Movement on Pain, Disability, and Kinematic Spinal Movements in Patients With Nonspecific Low Back Pain: A Randomized Placebo-Controlled Trial. *J Manipulative Physiol Ther*. 2018 Sep;38(6):365–74.

11. Moutzouri M, Billis E, Strimpakos N, Kottika P, Oldham JA. The effects of the Mulligan Sustained Natural Apophyseal Glide (SNAG) mobilisation in the lumbar flexion range of asymptomatic subjects as measured by the Zebris CMS20 3-D motion analysis system. *BMC Musculoskelet Disord*. 2008;9:1–9.

12. Waqqar S, Shakil-Ur-Rehman S, Ahmad S. McKenzie treatment versus mulligan sustained natural apophyseal glides for chronic mechanical low back pain. *Pakistan J Med Sci*. 2016;32(2):476–9.

13. Ajimsha MS, Al-Mudahka NR, Al-Madzhar JA. Effectiveness of myofascial release: Systematic review of randomized controlled trials. J Bodyw Mov Ther. 2018 Sep;19(1):102–12.

14. Ajimsha MS, Daniel B, Chithra S. Effectiveness of Myofascial release in the management of chronic low back pain in nursing professionals. *J Bodyw Mov Ther*. 2014;18(2):273–81.

15. Tozzi P, Bongiorno D, Vitturini C. Fascial release effects on patients with non-specific cervical or lumbar pain. *J Bodyw Mov Ther*. 2011;15(4):405–16.

16. Gauns S V, Gurudut P V. A randomized controlled trial to study the effect of gross myofascial release on mechanical neck pain referred to upper limb. *Int J Health Sci*.

2018;12(5):51–9.

17. Rezkallah SS, Abdullah GA. Comparison between sustained natural apophyseal glides (SNAG's) and myofascial release techniques combined with exercises in non specific neck pain. *Physiother Pract Res*. 2018;39(2):135–45.

18. Sadeghi R, Mosallanezhad Z, Nodehi-Moghadam A, Nourbakhsh MR, Biglarian A, Ezati K. The Reliability of Bubble Inclinometer and Tape Measure in Determining Lumbar Spine Range of Motion in Healthy Individuals and Patients. 2015 Oct 15;5(3):137-44.

19. Hefford C. The Patient-Specific Functional Scale: Validity, Reliability, and Responsiveness in Patients With Upper Extremity Musculoskeletal Problems. 2012;42(2):56–65.

20. Fritz JM, Irrgang JJ. A Comparison of a Modified Oswestry Low Back Pain Disability Questionnaire and the Quebec Back Pain Disability Scale. *Phys Ther*. 2001;81(2):776–88.

21. Hawker GA, Mian S, Kendzerska T, French M. Measures of adult pain: Visual Analog Scale for Pain (VAS Pain), Numeric Rating Scale for Pain (NRS Pain), McGill Pain Questionnaire (MPQ), Short-Form McGill Pain Questionnaire (SF-MPQ), Chronic Pain Grade Scale (CPGS), Short Form-36 Bodily Pain Scale (SF-BPS). *Arthritis Care Res*. 2011;63(SUPPL. 11):240–52.

Proforma

Screening Form

Name						
Age		Gender		Place		
Contact number						
Low back pain	specific			non specific		
Duration	< 3weeks		3 -12 weeks		> 12 weeks	
Height		Weight		BMI		

Included for study: - YES / NO

Data collection form

Participant number		Group				
Age		Gender		Place		
Contact number						
Duration	< 3weeks		3 -12 weeks		> 12 weeks	
Height		Weight		BMI		

Scales	Baseline data	Immediate effects	Post treatment effects
VAS score			
PSFS			
ODI			

Lumbar ROM assessment									
Flexion									
Extension									
Lateral flexion RIGHT									
Lateral flexion LEFT									
tightness if any									

Budget estimation

Items	Rate	Quantity	Total
Printing charges (informed consent, participant information sheet, data collection form and questionnaires)	27	75	2025
Mulligan Belt	500	1	500
Dissertation book	1500	4	6000
Stationaries and Miscellaneous (skin marking pens, travel)			1000
Total			9525

Appendix I

INFORMED CONSENT FORM

Study Title: The effects of Myofascial release (MFR) versus Mulligan Sustained Natural Apophyseal glides (SNAGs) in Nonspecific Low back pain: A Randomized Clinical Trail

Subject's initials:

Subject's name:

Date of birth/age:

1. I confirm that I have read and understood the information sheet dated__________ for the above study and have had the opportunity to ask questions.

2. I understand that my participation in the study is voluntary and that I am free to withdraw at any time, without giving any reason, without my medical care or legal rights being affected.

3. I understand that the sponsor of the controlled trial, others working on the sponsor's behalf, the ethics committee and the regulatory authorities will not need my permission to look at my health records both in respect of the current study and any further research that maybe conducted in relation to it, even if I withdraw from the trial. I agree to this access. However, I understand that my identity will not be revealed in any information released to third parties or published.

4. I agree not to restrict the use of any data or results that arise from this study provided such a use is only for scientific purpose(s).

5. I agree to take part in the above study.

Signature (or thumb impression) of the subject/legally acceptable representative:

________________ **Date: _________**

Signatory's name: ______________

Signature of the investigator: __________ **Date: _________**

Study investigator's name: ____________

Signature of the witness: ______________ **Date: _________**

Name of the witness: ________________

Appendix II

PARTICIPATION INFORMATION SHEET

TITLE OF STUDY

The effects of Myofascial release (MFR) versus Mulligan Sustained Natural Apophyseal glides (SNAGs) in Nonspecific Low back pain: A Randomized Clinical Trail

INVESTIGATOR

Vignesh Bhat P pursuing his Master's degree in Physiotherapy (Musculoskeletal sciences) at KMC Mangaluru

PURPOSE OF RESEARCH

To compare the immediate and short term effects of MFR and SNAGs in nonspecific low back pain.

PROCEDURE OF THIS STUDY

I understand that in this study, range of motion will be assessed, I have to complete the questionnaires given and then treatment will be provided. The study will be carried out for total 6 sessions.

BENEFITS OF THE STUDY

Results may act as substitute/alternative therapy to reduce pain, disability and improve lumbar range of motion

RISK AND DISCOMFORT

I understand that I may experience some discomfort while undergoing the evaluation and treatment. The entire procedure is safe and without any side effects

CONFIDENTIALITY

I understand that the medical information produced by this study will be kept confidential. If the data is used for publication in the medical literature or for teaching purpose; names and other identifiers will not be used without my permission.

REQUEST FOR MORE INFORMATION

I understand that, I may ask about any query about the study at any time to VIGNESH BHAT P at the following No. +919740698566 or email-id vinnu931995@gmail.com for further information regarding this study. Also he/she may contact Mrs Manisha P Shenoy, M.P.T, Guide, Assistant professor -senior scale, Department of Physiotherapy, Kasturba Medical College for any clarification and further study. The copy of consent form will also be given to me.

REFUSAL OR WITHDRAWAL OF PARTICIPANT

I understand that my participation is voluntary and that I may refuse to participate or may withdraw consent and discontinue participation in the study at any time.

INJURY STATEMENT

I understand that in case of any injury to me resulting directly from my participation in the study, medical treatment would be available, but no financial compensation would be provided for the same. I also understand that VIGNESH BHAT P may also terminate my participation in the study at any time without any reason.

I have explained to ___________________________the purpose of the research, the procedure, benefits and the risk factors associated with the study with my best ability.

Investigator: - ____________

Date: - ____________

I confirm that, VIGNESH BHAT P has explained the purpose of the research, the procedures involved in this study, the benefits and possible risk factor associated with the study. I have read and I understand this consent form, therefore I agree to give my consent to participation as a subject in this research project.

---------------------------- --------------------

Participant’s signature Date

---------------------------- ------------------------

Witness to signature Date

Appendix III

Visual Analog Scale

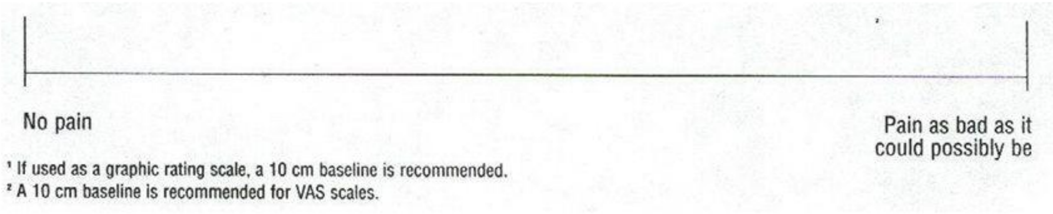

Appendix IV

Patient Specific Functional and Pain Scales (PSFS)

Name: **Date:**

Clinician Instructions: *Have patient complete after the history and before the exam*

Initial Assessment:
We want to know what 3 activities in your life you are unable to perform, or are having the most difficulty performing, as a result of your chief problem. Please list and score at least 3 activities that you are unable to perform, or are having the most difficulty performing, because of your chief problem

Follow Up Assessment:
When you were assessed on ________, you told us you had difficulty with the activities in the table below. Please score these activities that you told us previously you were unable to perform or were having difficulty performing because of your chief problem.

Scoring: Please score one number for each activity and for each date in the table below:

Unable to Perform Activity										Able to Perform Activity At Same Level As Before Injury/Problem
0	1	2	3	4	5	6	7	8	9	10

Activity	Date:	Date:	Date:	Date:	Date:
1.	Score (0-10)	Score (0-10)	Score (0-10)	Score (0-10)	Score (0-10)
2.	Score (0-10)	Score (0-10)	Score (0-10)	Score (0-10)	Score (0-10)
3.	Score (0-10)	Score (0-10)	Score (0-10)	Score (0-10)	Score (0-10)

Appendix V

Modified Oswestry Low Back Pain Disability Questionnaire[a]

This questionnaire has been designed to give your therapist information as to how your back pain has affected your ability to manage in everyday life. Please answer every question by placing a mark in the **one** box that best describes your condition today. We realize you may feel that two of the statements may describe your condition, but **please mark only the box that most closely describes your current condition.**

Pain Intensity
- ❑ I can tolerate the pain I have without having to use pain medication.
- ❑ The pain is bad, but I can manage without having to take pain medication.
- ❑ Pain medication provides me with complete relief from pain.
- ❑ Pain medication provides me with moderate relief from pain.
- ❑ Pain medication provides me with little relief from pain.
- ❑ Pain medication has no effect on my pain.

Personal Care (e.g., Washing, Dressing)
- ❑ I can take care of myself normally without causing increased pain.
- ❑ I can take care of myself normally, but it increases my pain.
- ❑ It is painful to take care of myself, and I am slow and careful.
- ❑ I need help, but I am able to manage most of my personal care.
- ❑ I need help every day in most aspects of my care.
- ❑ I do not get dressed, I wash with difficulty, and I stay in bed.

Lifting
- ❑ I can lift heavy weights without increased pain.
- ❑ I can lift heavy weights, but it causes increased pain.
- ❑ Pain prevents me from lifting heavy weights off the floor, but I can manage if the weights are conveniently positioned (e.g., on a table).
- ❑ Pain prevents me from lifting heavy weights, but I can manage light to medium weights if they are conveniently positioned.
- ❑ I can lift only very light weights.
- ❑ I cannot lift or carry anything at all.

Walking
- ❑ Pain does not prevent me from walking any distance.
- ❑ Pain prevents me from walking more than 1 mile. (1 mile = 1.6 km).
- ❑ Pain prevents me from walking more than 1/2 mile.
- ❑ Pain prevents me from walking more than 1/4 mile.
- ❑ I can walk only with crutches or a cane.
- ❑ I am in bed most of the time and have to crawl to the toilet.

Sitting
- ❑ I can sit in any chair as long as I like.
- ❑ I can only sit in my favorite chair as long as I like.
- ❑ Pain prevents me from sitting for more than 1 hour.
- ❑ Pain prevents me from sitting for more than 1/2 hour.
- ❑ Pain prevents me from sitting for more than 10 minutes.
- ❑ Pain prevents me from sitting at all.

Standing
- ❑ I can stand as long as I want without increased pain.
- ❑ I can stand as long as I want, but it increases my pain.
- ❑ Pain prevents me from standing for more than 1 hour.
- ❑ Pain prevents me from standing for more than 1/2 hour.
- ❑ Pain prevents me from standing for more than 10 minutes.
- ❑ Pain prevents me from standing at all.

Sleeping
- ❑ Pain does not prevent me from sleeping well.
- ❑ I can sleep well only by using pain medication.
- ❑ Even when I take medication, I sleep less than 6 hours.
- ❑ Even when I take medication, I sleep less than 4 hours.
- ❑ Even when I take medication, I sleep less than 2 hours.
- ❑ Pain prevents me from sleeping at all.

Social Life
- ❑ My social life is normal and does not increase my pain.
- ❑ My social life is normal, but it increases my level of pain.
- ❑ Pain prevents me from participating in more energetic activities (e.g., sports, dancing).
- ❑ Pain prevents me from going out very often.
- ❑ Pain has restricted my social life to my home.
- ❑ I have hardly any social life because of my pain.

Please complete questionnaire on other side.

Traveling

- ❑ I can travel anywhere without increased pain.
- ❑ I can travel anywhere, but it increases my pain.
- ❑ My pain restricts my travel over 2 hours.
- ❑ My pain restricts my travel over 1 hour.
- ❑ My pain restricts my travel to short necessary journeys under 1/2 hour.
- ❑ My pain prevents all travel except for visits to the physician / therapist or hospital.

Employment / Homemaking

- ❑ My normal homemaking / job activities do not cause pain.
- ❑ My normal homemaking / job activities increase my pain, but I can still perform all that is required of me.
- ❑ I can perform most of my homemaking / job duties, but pain prevents me from performing more physically stressful activities (e.g., lifting, vacuuming).
- ❑ Pain prevents me from doing anything but light duties.
- ❑ Pain prevents me from doing even light duties.
- ❑ Pain prevents me from performing any job or homemaking chores.

FOR OFFICE USE ONLY

Score: /50 x 100 = ____% points

Scoring: For each section the total possible score is 5: if the first statement is marked the section score = 0, if the last statement is marked it = 5. If all ten sections are completed the score is calculated as follows:

Example: 16 (total scored)
50 (total possible score) x 100 = 32%

If one section is missed or not applicable the score is calculated:

16 (total scored)
45 (total possible score) x 100 = 35.5%

Minimum Detectable Change (90% confidence): 10%points (Change of less than this amount may be attributed to error in the measurement.)

Name: ______________________________ **Date:** ______________

Source: Fritz JM, Irrgang JJ. A comparison of a modified Oswestry Low Back Pain Disability Questionnaire and the Quebec Back Pain Disability Scale. *Physical Therapy*. 2001;81:776-788.

[a]Modified by Fritz & Irrgang with permission of The Chartered Society of Physiotherapy, from Fairbanks JCT, Couper J, Davies JB, et al. The Oswestry Low Back Pain Disability Questionnaire. *Physiotherapy*. 1980;66:271-273.

Appendix VI

GANTT CHART

Title: Effects of Myofascial release versus Mulligan Sustained Natural Apophyseal Glides In Nonspecific Low Back Pain: A Randomized Clinical Trial																				
Primary Investigator : Vignesh Bhat P																				
Institution : Manipal Academy of Higher Education, KMC, Mangaluru																				
Time in months:	AUG 18	SEP 18	OCT 18	NOV 18	DEC 18	JAN 19	FEB 19	MAR 19	APR 19	MAY 19	JUN 19	JUL 19	AUG 19	SEP 19	OCT 19	NOV 19	DEC 19	JAN 20	FEB 20	MAR 20
Preparation of thesis protocol	■	■																		
Submission of protocol			■																	
Ethical committee approval				■																
To collect data					■	■	■	■	■	■	■	■	■	■	■	■	■			
To enter data					■	■	■	■	■	■	■	■	■	■	■	■	■			
1st departmental thesis progress review						■	■	■	■	■	■	■	■	■	■	■	■			
Analysis of collected data																		■		
Completion of thesis and preparation of manuscript for journal publication																			■	■
